# A Genetic Algorithm for Diploid Genome Reconstruction Using Paired-End Sequencing

**DOI:** 10.1371/journal.pone.0166721

**Published:** 2016-11-18

**Authors:** Chuan-Kang Ting, Choun-Sea Lin, Ming-Tsai Chan, Jian-Wei Chen, Sheng-Yu Chuang, Yao-Ting Huang

**Affiliations:** 1 Department of Computer Science and Information Engineering, National Chung Cheng University, Chiayi, Taiwan; 2 Agricultural Biotechnology Research Center, Academia Sinica, Taipei, Taiwan; 3 Biotechnology Center in Southern Taiwan, Academia Sinica, Tainan, Taiwan; 4 Institute of Biomedical Sciences, National Chung Hsing University, Taichung, Taiwan; Xiamen University, CHINA

## Abstract

The genome of many species in the biosphere is a diploid consisting of paternal and maternal haplotypes. The differences between these two haplotypes range from single nucleotide polymorphisms (SNPs) to large-scale structural variations (SVs). Existing genome assemblers for next-generation sequencing platforms attempt to reconstruct one consensus sequence, which is a mosaic of two parental haplotypes. Reconstructing paternal and maternal haplotypes is an important task in linkage analysis and association studies. This study designs and implemented HapSVAssembler on the basis of Genetic Algorithm (GA) and paired-end sequencing. The proposed method builds a consensus sequence, identifies various types of heterozygous variants, and reconstructs the paternal and maternal haplotypes by solving an optimization problem with a GA algorithm. Experimental results indicate that the HapSVAssembler has high accuracy and contiguity under various sequencing coverage, error rates, and insert sizes. The program is tested on pilot sequencing of a highly heterozygous genome, and 12,781 heterozygous SNPs and 602 hemizygous SVs are identified. We observe that, although the number of SVs is much less than that of SNPs, the genomic regions occupied by SVs are much larger, implying the heterozygosity computed using SNPs or *k*-mer spectrum may be under-estimated.

## Introduction

The release of next-generation sequencing (NGS) platforms, including 454 Life Sciences, Illumina Genome Analyzer, and Applied Biosystems SOLiD, have had a significant effect on many aspects of genomic research [1, 2]. Compared with traditional capillary-based Sanger sequencing, these NGS technologies are able to sequence tens of millions of reads at an affordable cost [3, 4]. Using these platforms, researchers have successfully assembled a number of genomes from microbial to mammalian scale in recent years. For example, the woodland strawberry genome was sequenced at a 39-fold coverage and over 95% of the genome was assembled using three NGS platforms [5]. The panda genome was the first mammalian genome sequenced and assembled using only the Illumina platform [6]. To understand the evolution of complex animal lives, the Genome 10K project aims to sequence the genomes of 10,000 vertebrates [7].

The objective of most genome sequencing projects aims to reconstruct a reference sequence from massive amount of short reads. Most genome assemblers adopt variations of the *de Bruijn* graph approach, which models the assembly problem as a search for an Eulerian path in the graph [8–10]. However, the performance of these short-read assemblers often deteriorates because of sequencing errors, repeats, and coverage variance [11]. To overcome the difficulty of assembling repeated regions, many researchers adopt paired-end sequencing to sequence both ends of larger read fragments (termed paired-end reads). These paired-end reads are used to further bridge assembled contigs into larger units called scaffolds [12, 13]. Finally, a second-round assembly can close the gaps within the scaffold [8].

In reality, the genome of most species in the biosphere is a diploid consisting of maternal and paternal haplotypes inherited from the parents. The differences between these two haplotypes range from small single-nucleotide polymorphisms (SNPs), small indels, to large-scale structural variations (SVs), including insertion, deletion, and inversion [14]. However, existing genome assemblers only attempt to reconstruct one consensus sequence, which is a mosaic of two parental haplotypes. Reconstructing paternal and maternal haplotypes is important for linkage analysis, association studies, and genomic imprinting [15]. Many computational approaches have been proposed for inferring the haplotypes via analysis of population linkage structure (called phasing). But these methods assumes a reference genome is available and sufficient genomes are sequenced, while most *de novo* sequencing projects only sequence one genome. This paper focuses on haplotype reconstruction in *de novo* sequencing when only one genome is deeply sequenced.

Existing methods can be classified into three categories. First, a number of methods can identify heterozygous SNPs/SVs (differed between parental haplotypes) using coverage analysis after mapping reads onto a reference genome (e.g., SAMtools). But the allele linkage of variations along each parental haplotype is not resolved. The second category of methods directly reconstruct the paternal/maternal sequences from short reads [16], which simultaneously solve the genome assembly and haplotype reconstruction problems. However, this strategy reduces the flexibility for taking advantages of novel sequencing technologies (e.g., PacBio sequencing) and of algorithmic improvement (e.g., paired de Bruijn graph). The third type of methods independently solve the genome assembly and haplotype reconstruction problems, providing the flexibility for using newly-developed assemblers. After a consensus (mosaic) sequence is assembled, the parental haplotypes are reconstructed by analysis of allele linkage across heterozygous loci [17, 18]. This paper belongs to the third category. The Craig Ventor Genome was the first diploid genome assembled using this way [17]. The parental haplotypes were assembled by joining overlapping (single-end) reads that span two or more SNPs. But it does not consider variations other than SNPs. Nowadays, paired-end sequencing is widely used in most sequencing projects and contains rich information for identifying various types of genetic variations (e.g., identification of SVs) [2, 19, 20], which can serve as a better resource for reconstructing haplotypes.

This study presents the design and implementation of a novel method called the HapSVAssembler for the *de novo* assembly of paternal and maternal haplotypes based on paired-end sequencing. The proposed method first builds a consensus sequence, identifies the heterozygous loci of SNPs/SVs, and reconstructs the paternal and maternal haplotypes by solving an optimization problem with a genetic algorithm (GA). The experimental results indicate that this method has high accuracy and contiguity under various sequencing coverage rates, error rates, and insert sizes. The program is tested on a pilot sequencing of a highly heterozygous genome and resontructs paternal and maternal sequences composed of heterozygous SNPs and hemizygous SVs.

## Method

**Fig 1:**
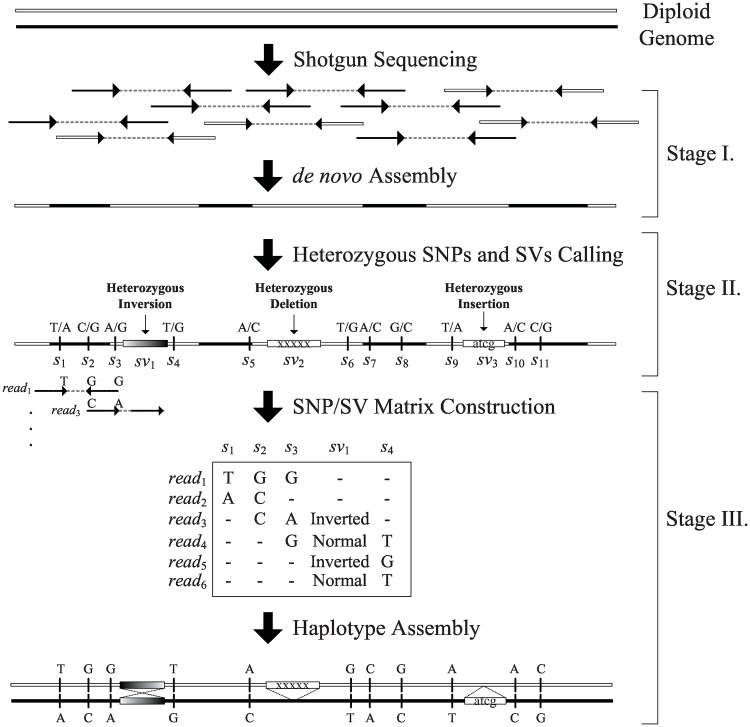
Overview of HapSVAssembler. Overview of HapSVAssembler. Stage I: Using *de* *novo* assembler to reconstruct a reference genome; Stage II: Using a reference genome assembled in Stage I, we can find SNPs and heterozygous SVs; Stage III: Two consensus haplotypes can be reconstructed from the SNP/SV matrix.

Fig 1 shows a flowchart of the HapSVAssembler and the detailed software components can be found in S1 Fig. Given a set of paired-end reads, the program first constructs a set of consensus contigs by integrating *de Bruijn* graph and overlap graph assemblers for assembly in low- and high-coverage regions. In the second stage, the program aligns all reads to the assembled contigs and identifies heterozygous loci, including SNPs, insertions, deletions, and inversions. In the final stage, the program extracts reads spanning at least two heterozygous loci, divides reads into paternal and maternal groups, and reconstructs the paternal and maternal haplotypes by solving an NP-hard problem called constrained minimum error correction (CMEC). This study also proposes a novel GA for the CMEC problem.

### Stage I: Construction of a Reference Consensus Sequence

**Fig 2:**
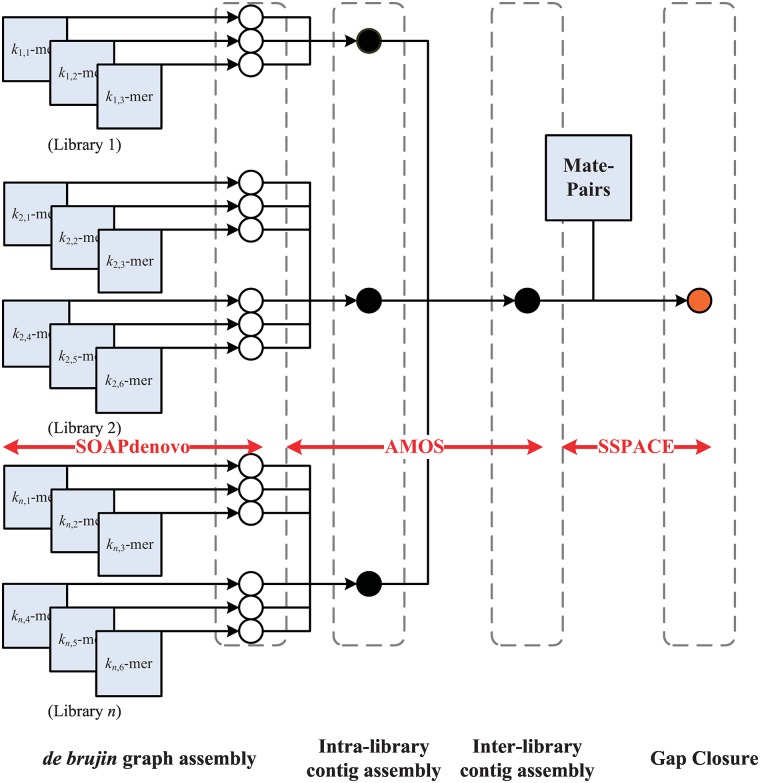
Flowchart of hybrid *de novo* assembly approach. The flowchart of the *de novo* assembly using hybrid approach with.

The consensus sequence can be first built using any existing assembler (e.g., SOAPdenovo, ABySS). As each assembler has its own strength and weakness, we present a hybrid pipeline used internally for typical Illumina sequencing. Existing short-read assemblers (e.g., SOAPdenovo) must break down the reads into fixed-length *k*-mers to build a *de Bruijn* graph, which implies a minimum overlap length between reads. In high-coverage regions, larger *k*-mers are good for reducing the graph complexity and improving assembly accuracy. Smaller *k*-mers are more appropriate for low-coverage regions because of the insufficient overlap between reads. Consequently, we uses a *de Bruijn* graph assembler to assemble reads into contigs using multiple *k*-mers (e.g., *k* = 25∼49) to adapt to the coverage variance across the entire genome. The second phase merges the contigs consistently assembled in multiple *k*-mers into meta-contigs by using an overlap-graph assembler (called AMOS [21]). This is because overlap-graph assemblers do not break contigs into smaller *k*-mers to build a graph. This merging process discards the singleton contigs assembled in only one *k*-mer, and attempts to elongate the more accurate contigs from larger *k*-mers and remove possible misassembled contigs from smaller *k*-mers. The third phase links these meta-contigs into scaffolds by using paired-end or mate-pair reads through SSPACE [12]. Finally, the assembly gaps within scaffolds are closed by unused reads using GapCloser [8]. This workflow is shown in Fig 2. The users may choose the best assembly pipeline for distinct sequencing platforms (e.g., SMRT for PacBio).

### Stage II: Identification of Heterozygous SNPs and SVs within a Diploid Genome

The assembled contigs in Stage I form a mosaic sequence consisting of paternal and maternal haplotypes. The genomic variants between these two haplotypes include small-scale SNPs/indels to large-scale SVs (e.g., insertions, deletions, and inversions). The small-scale variants can be identified by analyzing the read alignment output (i.e., gaps or mismatches). Conversely, the analysis of paired-end reads often reveals large-scale SVs [22–24]. The detectable genomic variants must be heterozygous between the paternal and maternal haplotypes because at least two distinct alleles appear at the same locus. Standard SNP/indel callers (e.g., SAMTool or GATK) provide sufficient information (in SAM and VCF standard) to distinguish reads carrying different alleles, which is necessary for subsequent haplotype assembly. However, existing SV callers (e.g., Breakdancer, MoDIL, or VariationHunter) cannot supply the information required to distinguish reads for SV or non-SV haplotypes, and the accuracies of reported SVs and boundaries are unsatisfactory [19, 20]. Therefore, the HapSVAssembler invokes BWA to align the reads onto the assembled contigs, and uses SAMTools to identify the coordinate/alleles of each heterozygous SNP and indel. For large SVs, a novel SV detection module not only outputs accurate SV and boundary values, but also distinguishes reads spanning SV or non-SV haplotypes.

**Fig 3:**
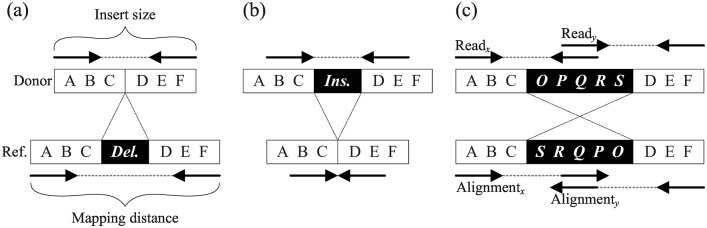
Signatures of discordant reads implying SVs. (a) The mapping distance of a deletion event is larger than insert size; (b) The mapping distance of an insertion event is smaller than insert size; (c) The orientations of both ends of a read spanning an inversion breakpoint will turn to (+, +) (Read_*x*_) or (−, −) (Read_*y*_).

The SV detection module captures two important SV signatures: discordant reads and breakpoint reads. Theoretically, the mapping distances of both ends of a paired-end read from a non-SV region (called concordant reads) should be roughly equal to the expected insert size, and the orientations of both ends should be (+, −) or (−, +). However, for paired-end reads across large insertions or deletions (called discordant paired-end reads), the mapping distances between both ends are significantly smaller or larger, respectively, than the expected insert size (Fig 3(a) and 3(b)). For paired-end reads spanning boundaries of an inversion, the orientations of both ends change to (+, +) or (−, −) (Fig 3(c)). Genomic regions containing excess discordant reads with aberrant mapping distances or orientations are indicative of SVs. However, the SV boundaries identified solely by discordant reads are often imprecise because of the variance of the insert size. Thus, the proposed SV detection module integrates discordant reads and breakpoint reads to delineate precise boundaries for each type of SV, as described in the following subsections.

#### SV Detection via Discordant Reads

**Fig 4:**
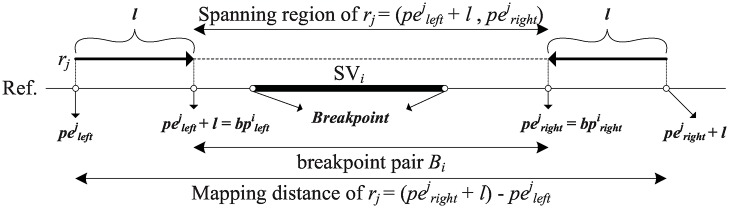
Identification of insertions or deletions. A discordant read *r*_*j*_ is mapped on the reference with two mapping locis, $pe_{left}^{j}$ and $pe_{right}^{j}$. The spanning region of *r*_*j*_ is from $\left(pe_{left}^{j}+l\right)$ to $pe_{right}^{j}$. And the potential breakpoint pair of SV_*i*_ is initialized from $pe_{left}^{j}+l$ to $pe_{right}^{j}$.

This section first introduces the notations used in this study. Suppose that the coordinate of the breakpoint pair of a potential SV_*i*_ is denoted by $B_{i}=\left(bp_{left}^{i},bp_{right}^{i}\right)$. Denote the two mapping loci of the *j*-th paired-end read *r*_*j*_ as $pe_{left}^{j}$ and $pe_{right}^{j}$. The spanning region of *r*_*j*_ ranges from ($pe_{left}^{j}+l$) to $pe_{right}^{j}$, where *l* is the read length. The mapping distance of *r*_*j*_ is notated by $md_{j}=\left(pe_{right}^{j}-pe_{left}^{j}+l\right)$ (Fig 4). Assume that the mapping distances of all paired-end reads follow a normal distribution with mean *μ* and standard deviation *sd* [25].

**Fig 5:**
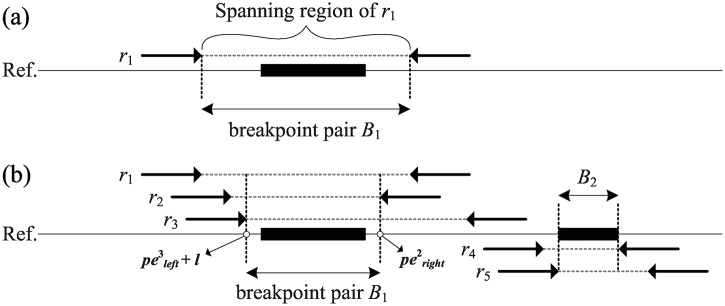
Identification of clustered insertion/deletion evidicnes. (a) The breakpoint pair of potential SV_1_ is defined by the spanning region of the first discordant read *r*_1_; (b) *r*_2_ and *r*_3_ are joined into *C*_1_ due to the overlapping with *B*_1_ in (a). *r*_4_ does not overlap with *B*_1_; therefore, a new cluster *C*_2_ is created.

The proposed method detects large deletions or insertions by searching for clusters of discordant reads with significantly larger or smaller mapping distances. Define a discordant read with aberrant mapping distance as ∣*md*_*j*_ − *μ*∣ > 2*sd*. For ease of explanation, this study focuses on the detection of large deletions. However, large insertions are found in a similar way. The discordant reads are sequentially processed according to the coordinate of their mapping loci. Each discordant read is assigned to a cluster *C*_*i*_ of discordant reads, which may imply a potential SV_*i*_. An initial cluster *C*_1_ is created if supported by the first discordant read *r*_1_, and the SV type (insertion or deletion) of SV_1_ is recorded according to the mapping distance. The size of SV_1_ is computed by ∣*md*_1_ − *μ*∣. The inferred breakpoints of SV_1_ are initially set to the spanning region of *r*_1_, such that $B_{1}=\left(bp_{left}^{1},bp_{right}^{1}\right)=\left(pe_{left}^{1}+l,pe_{right}^{1}\right)$ (Fig 5(a)). The remaining discordant reads are assigned to an existing cluster *C*_*i*_ only if their SV type is identical and the spanning region overlaps the existing breakpoint pair *B*_*i*_. Otherwise, a new cluster is created (Fig 5(b)). When assigning a discordant read *r*_*j*_ to an existing cluster *C*_*i*_, re-compute the SV size by $\frac{\sum_{r_{j}\in C_{i}}|md_{j}-\mu |}{|C_{i}|}$, and tighten the breakpoint pair *B*_*i*_ by intersecting the spanning region of *r*_*j*_, such that $B_{i}=\left(bp_{left}^{i},bp_{right}^{i}\right)\;\cap \;\left(pe_{left}^{j}+l,pe_{right}^{j}\right)$. Recursively execute this clustering procedure until all discordant reads with an aberrant mapping distance are assigned to a cluster.

**Fig 6:**
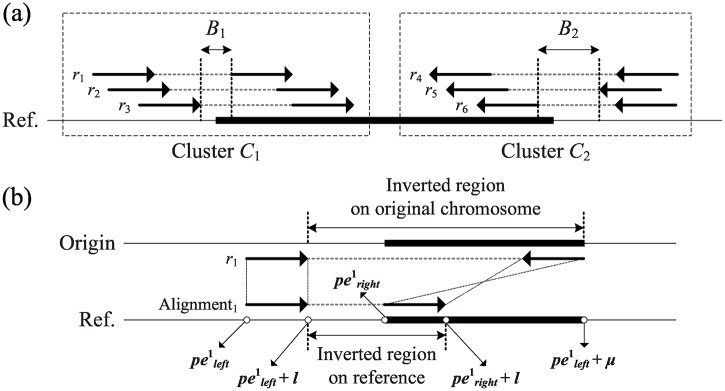
Identification of inversions. (a) Two breakpoints from the same inversion are broken into two clusters *C*_1_ and *C*_2_ owing to the intersection strategy; (b) The longer inverted region has been observed on the original chromosome; therefore, the final inverted region $read_{1}^{invert}$ of *r*_1_ is ranged from $\left(pe_{right}^{1}+l\right)$ to $\left(pe_{left}^{1}+\mu \right)$ on reference.

The identification of an inversion mainly relies on paired-end reads with aberrant orientations. A read with a (+, +) aberrant orientation implies that its right end is within the inversion and the left end is outside the inversion. Similarly, a read with a (−, −) aberrant orientation has its left end within the inversion and right end outside inversion. Using a clustering procedure similar to that used in deletion/insertion detection, the left and right breakpoints of an inversion can be determined by recursively clustering each discordant read with the same type of aberrant orientation (Fig 6(a)). Each inversion induces two discordant clusters, which is found to be confused by clusters of other inversions in practice. To identify the two clusters associated with each inversion, compute the maximum extent of the possible inverted region implied by each read, such that two clusters belonging to the same inversion can be associated. The maximum inverted region of a discordant read *r*_*j*_, which is denoted as $read_{j}^{invert}$, is formulated as follows:

The mapping distances between two ends of a paired-end read are definitely smaller than the inversion size. Therefore, choose the maximum possible value to represent the maximum extent of the inverted region (Fig 6(b)). This approach guarantees that the overlap between any two clusters belonging to the same inversion will be identified.

**Fig 7:**
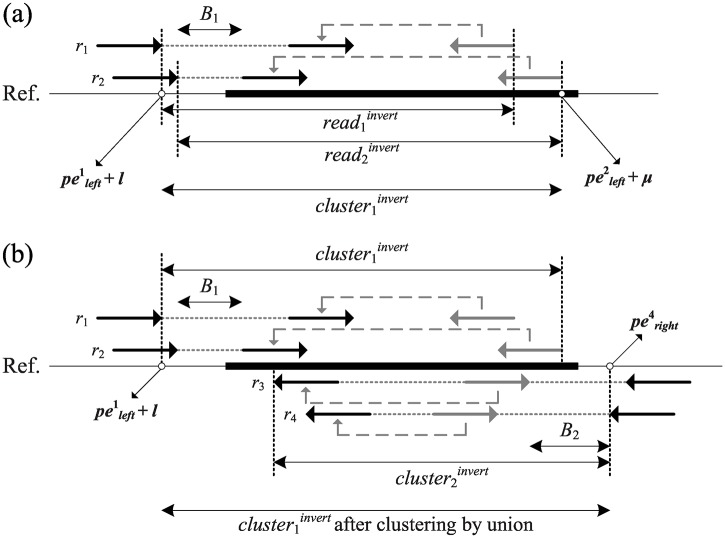
Identification of clustered inversion evidences. (a) The solid gray arrow represents the beginning loci on the original chromosome, and its mapping location on the reference is pointed by a dotted arrow. The maximum inverted region of a cluster *C*_1_ can be determined by union the inverted regions from all its supporting reads; (b) *C*_1_ and *C*_2_ can be clustered together using the union operator to join $cluster_{1}^{invert}$ and $cluster_{2}^{invert}$.

Let $cluster_{i}^{invert}$ be one cluster of discordant reads; that is, $cluster_{i}^{invert}={⋃}_{r_{j}\in C_{i}}read_{j}^{invert}$ (Fig 7(a)). Subsequently, the two clusters *C*_*i*_ and *C*_*j*_ can be merged if $(cluster_{i}^{invert}\;⋂\;cluster_{j}^{invert})\notin \emptyset$, and the merged inverted region is updated to ($cluster_{i}^{invert}\;⋃\;cluster_{j}^{invert})$ (Fig 7(b)). After this union procedure, two clusters belonging to the same inversion combine into a larger cluster.

$$\begin{array}{c} read_{j}^{invert}=\{\begin{array}{cc} \left(pe_{left}^{j}+l,\max\left\{\left(pe_{right}^{j}+l\right),\left(pe_{left}^{j}+\mu \right)\right\}\right) & \text{if}\;r_{j}\in \left(+,+\right)\\ \left(\min\left\{pe_{left}^{j},\left(pe_{right}^{j}+l-\mu \right)\right\},pe_{right}^{j}\right) & \text{if}\;r_{j}\in \left(-,-\right) \end{array} \end{array}$$ (1)

#### SV Boundary Refinement via Breakpoint Reads

**Fig 8:**
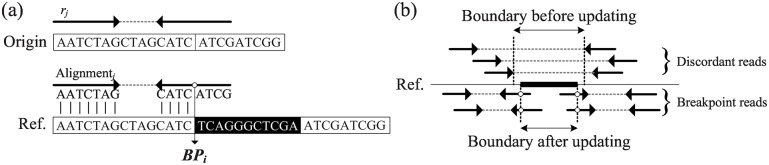
Illustration of breakpoint reads across SV boundaries. (a) A breakpoint Read *r*_*j*_ whose right end matches perfectly first 4 nucleotides whether the remainder bases are mismatched with the reference. The guessing breakpoint can be inferred at the 4th base of the right end on *r*_*j*_; (b) The actual breakpoints of SV can be determined by breakpoint reads.

The SV boundaries identified by discordant reads are often imprecise. To refine the SV boundaries, the HapSVAssembler identifies the reads spanning SV boundaries (called breakpoint reads) by parsing the SAM alignment results. These breakpoint reads often leave a footprint of continuous unmapped or mismatched positions in SAM alignment (e.g., 40M40S for an 80 bp read). This is because conventional short-read alignment algorithms (e.g., BWA) do not open large gaps for splitting reads across large variations. Instead, these breakpoint reads are often partially aligned to the reference genome because the read fragments within SV are often unmappable or mismatched (Fig 8(a)). Denote the SV boundary implied by the *j*th breakpoint read as *BP*_*j*_. For any two breakpoint reads implying the same SV boundary (i.e., *BP*_*j*_ = *BP*_*i*_), maintain a counter recording the frequency of breakpoint reads at this locus. Thereafter, use these breakpoint reads to update the breakpoint pair *B*_*i*_ of each identified potential SV if the implied breakpoint is within $B_{i}=\left(bp_{left}^{i},bp_{right}^{i}\right)$ (Fig 8(b)). The breakpoint reads are ignored if they do not overlap with any SV candidates.

#### Analysis of False Discovery Rates

Integrating discordant and breakpoint reads for calling SVs produces a relatively low error probability. The insert size of paired-end reads (of the same library) approximates a normal distribution [25], and the requirement of aberrant mapping distances for discordant reads (i.e., |*md*_*j*_ − *μ*| > 2*sd*) implies a confidence interval greater than 95% and error probability less than 5%. In practice, we require at least *s* discordant reads for calling an SV, leading to an error probability of (0.05)^*s*^. Thus, the default minimum requirement of five discordant reads has an error probability of ≈2 × 10^−4^. In addition, the error probability of a breakpoint read with length *l* can be computed via a binomial distribution. Let the sequencing error rate be *e*, and the number of matching positions of the *j*-th breakpoint read be *n*_*j*_. The error probability of requiring at least *k* breakpoint reads for calling an SV is ∏j=1k(lnj)(1-e)njel-nj. In reality, with the typical error rate of approximately 1% on the Illumina platform, an 80 bp read length, at least 40 bp matches and two breakpoint reads, the error probability of SVs miscalled by HapSVAssembler is ∏j=12(8040)(0.99)400.0140≈5.17×10-115. Thus, the default minimum requirement of at least five discordant reads or at least two breakpoint reads has an error probability of less than 2 × 10^−4^ or 5.17 × 10^−115^, respectively.

### SNP/SV Matrix Construction and Haplotype Block Partition

#### SNP/SV Matrix Construction

**Fig 9:**
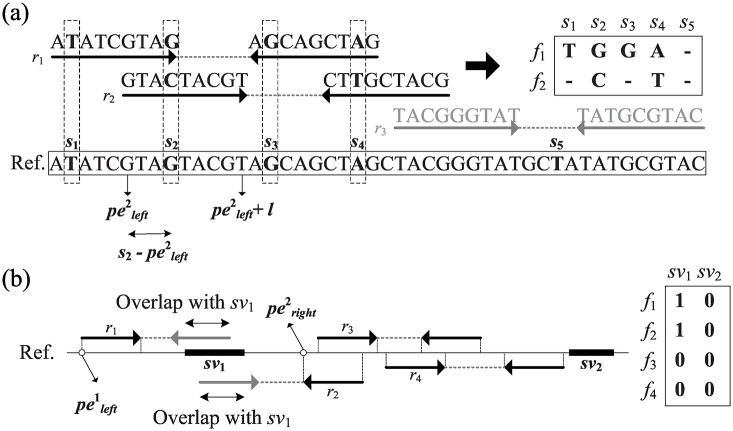
Illustration of converting paired-reads to SNP matrix and SV matrix. (a) Paired-end read *r*_1_ and *r*_2_ both contain SNPs but *r*_3_ does not, therefore, *r*_1_ and *r*_2_ can be successfully converted to read fragment *f*_1_ and *f*_2_ respectively. SNP *s*_2_ is covered by *r*_2_, and the allele at *s*_2_ can be obtained by the 4-th $\left(s_{2}-pe_{left}^{2}\right)$ nucleotide on *r*_2_; (b) Single-end mapped read *r*_1_ and *r*_2_ whose unmapped ends are overlapping with *sv*_1_ (e.g., a deletion), both of $M_{1,1}^{SV}$ and $M_{2,1}^{SV}$ can be assigned by 1.

Given a set of SNPs and SVs, the HapSVAssembler attempts to identify reads carrying distinct alleles (e.g., nucleotide or inversion orientation) and convert them into an *m* by *n* SNP/SV matrix *M*, where *m* is the number of read fragments, and *n* is the total number of SNP and SV sites. This study defines an *m* by *n*_*snp*_ sub-matrix *M*^*SNP*^ from *M*, where *n*_*snp*_ is the total number of SNPs. Assume that *s*_*j*_ is the *j*th SNP locus and the set of SNPs on the *i*th paired-end read is defined as a read fragment *f*_*i*_ if and only if $\exists \;1\leq j\leq n_{snp}\;\left(\left(pe_{left}^{i}\leq s_{j}\leq pe_{left}^{i}+l\right)\vee \left(pe_{right}^{i}\leq s_{j}\leq pe_{right}^{i}+l\right)\right)$. The term $M_{i,j}^{SNP}$ means that the allele at SNP *s*_*j*_ of fragment *f*_*i*_ is represented by {A, C, G, T, –}, where ‘–’ denotes the unknown base. The term $M_{i,j}^{SNP}$ can be assigned by the *k*th nucleotide on *r*_*i*_, where *k* is the distance from $pe_{left}^{i}$ or $pe_{right}^{i}$ to *s*_*j*_ if $\left(pe_{left}^{i}\leq s_{j}\leq pe_{left}^{i}+l\right)$ or ($\left(pe_{right}^{i}\leq s_{j}\leq pe_{right}^{i}+l\right)$, respectively (Fig 9(a)). Conversely, an *m* by *n*_*sv*_ sub-matrix *M*^*SV*^ represents the association between fragments and SVs, where *n*_*sv*_ is the number of discovering heterozygous SVs. Assume that *sv*_*j*_ is the *j*th SV location, $M_{i,j}^{SV}$ represents the SV type of *sv*_*j*_ that fragment *f*_*i*_ covers, and $M_{i,j}^{SV}$ is represented by {0: no SV, 1: deletions, 2: insertions, 3: inversions}. A single-end mapped read indicates that the unmapped end is likely to be located in a heterozygous SV (Fig 9(b)). If *r*_*i*_ is left-end mapped to the reference, $M_{i,j}^{SV}$ can be defined as follows.

$$\begin{array}{c} M_{i,j}^{SV}=\{\begin{array}{cc} \text{type}\;\text{of}\;sv_{j} & \text{if}\;\left(pe_{left}^{i}+l,pe_{left}^{i}+\mu \right)\cap \left(bp_{left}^{j},bp_{right}^{j}\right)\notin \emptyset \\ 0 & \text{otherwise} \end{array} \end{array}$$ (2)

Similarly, if *r*_*i*_ is right-end mapped to the reference,

$$\begin{array}{c} M_{i,j}^{SV}=\{\begin{array}{cc} \text{type}\;\text{of}\;sv_{j} & \text{if}\;\left(pe_{right}^{i}+l-\mu ,pe_{right}^{i}\right)\cap \left(bp_{left}^{j},bp_{right}^{j}\right)\notin \emptyset \\ 0 & \text{otherwise} \end{array} \end{array}$$ (3)

#### Haplotype Blocks Partition for Parallel Computation

**Fig 10:**
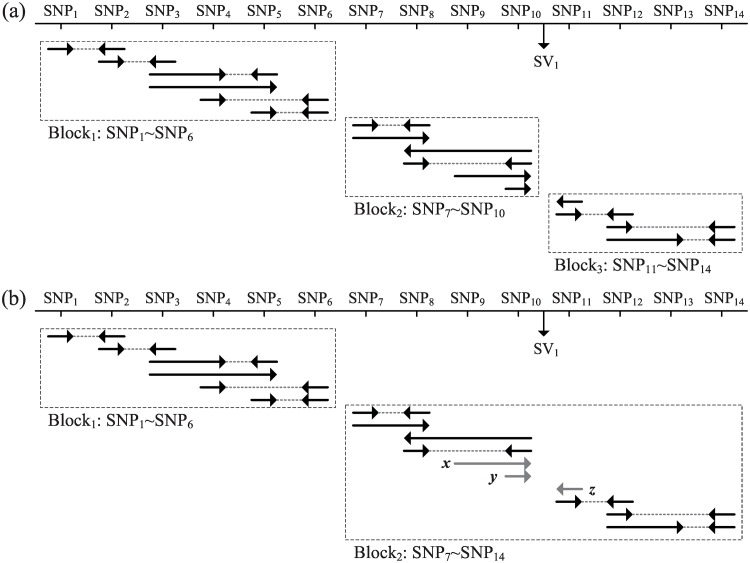
Illustration of extended Haplotype blocks via heterozygous SVs. One end is represented by a solid arrow and two ends from the same read are connected by a dotted line. There is a heterozygous SV_1_ between SNP_10_ and SNP_11_. (a) Without considering SVs, the entire haplotype will be broken into three haplotype blocks; (b) In our approach, Block_2_ and Block_3_ in (a) are merged by bridging read *x*, *y* in Block_2_ and bridging read *z* in Block_3_ that indicate heterozygous SV_1_.

In reality, the paired ends may not overlap continuously because of low-coverage or sequencing gaps, leading to a number of isolated overlapping groups called haplotype blocks. The haplotype assembly within a haplotype block is independent of other blocks (Fig 10(a)). Therefore, this study uses a simultaneous haplotype assembly through the parallel computation of multithreads (OpenMP) to significantly improve assembly efficiency. Because this approach simultaneously assembles multiple types of genomic variants (e.g., SNPs, insertions, and deletions), the resulting haplotype blocks are larger than those of methods based on SNPs alone. This is because a heterozygous SV can bridge two distinct haplotype blocks if they are spanned by any SV read. Therefore, two adjacent blocks can be merged if bridging reads in both adjacent blocks indicate the same SV (Fig 10(b)).

### Haplotype Assembly within a Haplotype Block

#### Constrained MEC Formulation

This haplotype assembly within a haplotype block is formulated into a constrained version of the MEC problem, which aims to partition reads into two consensus haplotypes with minimum error corrections, requiring reads carrying identical SV signatures are assigned to the same haplotype. The optimal solution of the CMEC for error-free reads is zero because there should be no conflict between read fragments and corresponding consensus haplotypes. However, sequencing errors make it difficult to find a partition without conflicts. Hence, the CMEC problem attempts to divide a partition of reads into two groups to minimize the number of conflicts. In addition, we observed that reads carrying identical SV signatures almost come from the same haplotype. Therefore, the reads having the same SV signatures are used as constraints during read partition. Specifically, if an SNP/SV matrix *M* and *H* = (*h*_0_, *h*_1_) represents the consensus haplotype pair, the number of error correction between the *i*th read fragment *f*_*i*_ and consensus haplotype *h*_*l*_ at the SNP site *s*_*k*_ is defined as

$$\begin{array}{c} d\left(M_{i,k}^{SNP},h_{l,k}\right)=\{\begin{array}{cc} 1 & \text{if}\;M_{i,k}^{SNP}\neq h_{l,k}\neq -\\ 0 & \text{otherwise} \end{array} \end{array}$$ (4)

Therefore, the total error correction numbers between read *f*_*i*_ and haplotype *h*_*l*_ is defined as $D\left(f_{i},h_{l}\right)=\sum_{k=1}^{n_{snp}}d(M_{i,k}^{SNP},h_{l,k})$. Furthermore, *P* = (*p*_0_, *p*_1_) stands for a possible partition of all fragments, and all fragments *f*_*i*_ ∈ *p*_*l*_ will construct the consensus haplotype *h*_*l*_. The CMEC problem can be formulated as follows:

$$\begin{array}{cc} \text{minimize} & \sum_{l=0}^{1}\sum_{f_{i}\in p_{l}}D(f_{i},h_{l})\\ \text{subject to} & \left\{f_{i},f_{j}\right\}\subseteq p_{l}\text{if}\;M_{i,k}^{SV}=M_{j,k}^{SV}\neq 0,\\ & l=\{0,1\},\\ & 1\leq i,j\leq m,\\ & 1\leq k\leq n_{sv}. \end{array}$$

The CMEC problem is a generalized version of the NP-hard MEC problem [26, 27], and is therefore also NP-hard. The proposed method uses the GA to address small instances of the MEC problem [28]. However, existing GA frameworks are inadequate for solving the CMEC problem because the search space is exponential to the enormous number of reads in practical NGS platforms. Although not shown in this paper, the solution quality and running time of the original GA are both far from practical use. Therefore, this study presents a GA framework with novel initialization and mutation schemes to solve the CMEC problem in a large solution space.

#### A Genetic Algorithm for Solving the Constrained MEC Problem

Genetic algorithm (GA) simulates the mechanisms of natural evolution, such as selection, crossover, and mutation, to evolve the candidate solutions to their optimum values. The effectiveness of this approach has been validated in numerous search and optimization problems. GA represents candidate solutions as chromosomes. Instead of using a single search point, GA conducts a global search through a set (population) of chromosomes. The fitness function evaluates the quality (fitness) of chromosomes. The evolution in the GA begins with the population initialization. GA then initiates the reproduction process. The selection operator first picks two chromosomes from the population as parents. Next, the GA performs crossover on these two parents to reproduce their offspring. Some genes are altered by the mutation operator for diversity. Implementing a Survival of the Fittest function, the survivor operator draws the fittest chromosomes out of the union of parent and offspring populations, and these chosen chromosomes constitute the population for the next generation.

To reduce the computational effort in stochastic search, this study incorporates a local search into the initialization and mutation operators of the GA to improve the search efficiency and solution quality. The experimental results in the next section confirm that this new GA can achieve better solutions in a shorter time than a standard GA. The following paragraphs present more details about the proposed GA, where the deatiled GA parameters are listed in Table 1.

**Table 1:** Parameter setting in GA.

Operations/Parameters	Setting
Representation	Binary string
Initialization	Heuristic
Population size	10
Crossover	Uniform
Crossover rate	100%
Number of offspring	10
Mutation rate	In error list: 80%; otherwise: $\frac{1}{m^{\prime }}$
Parent selection	two-tournament
Survivor selection	Replace worst
Termination	5 generations

**I. Representation**

**Fig 11:**
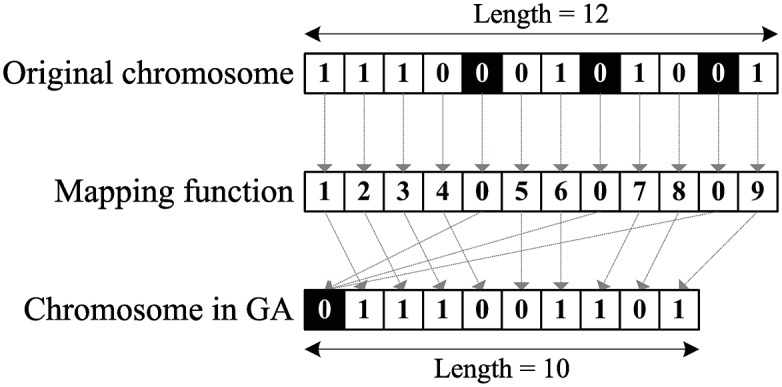
Reducing GA chromosome length via a mapping function. In original chromosome, there are three fragments indicate the same SV (mark by black). The mapping function indicates the exact index of the chromosome in GA and three SV-associated fragments will point to the same index (index 0).

Because all read fragments should must be partitioned into two disjoint sets, the proposed GA represents a chromosome as a binary string over {0, 1}, where 0 and 1 respectively stand for the two sets. Considering the constraints of the CMEC, read fragments carrying the same SV allele $\left(M_{i,k}^{SV}=M_{j,k}^{SV}\neq 0\right)$ must be forcibly partitioned into the same set, i.e., {*f*_*i*_, *f*_*j*_} ⊆ *p*_*l*_. Therefore, we use only one bit to represents the set of read fragments indicating the same SV, and the chromosome length is reduced from *m* to $m^{\prime }=\left(m-\sum_{M_{i,k}^{SV}\neq 0}1+n_{sv}\right)$. A mapping function can transform the original chromosome to a reduced chromosome in constant time (Fig 11).

**II. Population Initialization**

**Fig 12:**
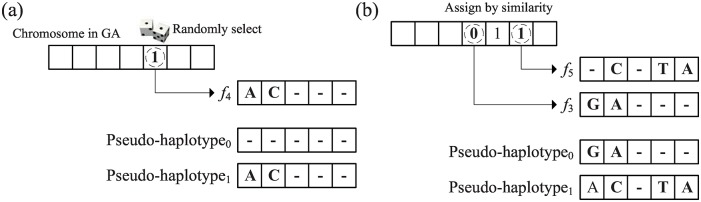
Heuristic population initialization for GA chromosomes. (a) The set of starting fragment *f*_4_ is randomly set as 1, and we will update the pseudo-haplotype_1_; (b) The pseudo-haplotypes are extended from *f*_3_ and *f*_5_, and the set of *f*_3_ and *f*_5_ is determined by the similarity.

To generate an initial partition (chromosome) *P*^0^, randomly select a read fragment *f*_*s*_ as a starting point, where 2 ≤ *s* ≤ *m*′ − 1. All read fragments are sorted according to their mapping coordinates. A random set is assigned to *f*_*s*_ at the beginning, and the pseudo (consensus) haplotype corresponding to this set is also updated by the alleles on *f*_*s*_ (Fig 12(a)). The pseudo haplotype is then sequentially updated by reads flanking *f*_*s*_ in both directions. For each flanking read *f*_*i*_, compute the similarity between *f*_*i*_ and the two pseudo-haplotypes, and then greedily assign *f*_*i*_ as follows (Fig 12(b)).

After assigning a read fragment to a set, the allele of the corresponding pseudo-haplotype may be updated to maintain only major alleles. This initialization process repeats until the population of chromosomes is generated. Simulation results show that this heuristic initialization can construct solutions relatively close to the optimum because the sequencing error rate is often not high and thus the number of conflicting reads is relatively low in practice. This randomized greedy initialization also generates possible partitions implied by the conflicting reads only. This approach greatly reduces the running time of the original GA, which randomly generates partitions of all reads.

**III. Fitness Evaluation**

The consensus haplotypes must be generated before evaluating the fitness value of a partition. Define $N_{allele}^{k}\left(l\right)=\left(\;\sum_{f_{i}\in p_{l};M_{i,l}^{SNP}=allele}1\;\right)$ as the number of fragments carrying *allele* at *s*_*k*_ in *p*_*l*_, where *allele* ∈ {A, C, G, T}. The *k*th site of the consensus haplotype is defined by

$$\begin{array}{c} h_{l,k}=\underset{allele}{\mathrm{argmax}}\;N_{allele}^{k}\left(l\right) \end{array}$$

To construct a consensus haplotype at each site from the fragments, greedily select the major allele that is supported from the majority. The fitness value of a partition *P* is defined as

$$\begin{array}{c} F\left(P\right)=\sum_{l=0}^{1}\sum_{f_{i}\in p_{l}}D(f_{i},h_{l}). \end{array}$$

**IV. Genetic Operators**

The proposed GA adopts the two-tournament selection operator in view of its recognized good performance. This selection operator chooses the better of two randomly selected chromosomes as a parent. The selection procedure iteratively runs twice to obtain a pair of parents for subsequent crossover operation.

The crossover operation exchanges and recombines the genetic information of both parents. The GA employs the widely used uniform crossover, which randomly determines each offspring gene from either parent. This mutation operation slightly changes the composition of the offspring. This paper devises a mutation operator based on the bit-flip mutation that flips (i.e., 0 → 1, 1 → 0) genes with a predefined probability called the mutation rate *p*_*m*_. The proposed mutation also uses an error list of a partition to record the index of fragments that conflicts with the consensus haplotype. The fragments in the error list require a mutation rate exceeding 0.8 to be flipped into the other set; those that remain have a lower mutation rate $\frac{1}{m^{\prime }}$.

Finally, to achieve good solutions from the mix of parent and offspring populations over the course of GA evolution, solutions with higher fitness values are selected to survive to the next generation. The termination criterion is set to five generations, at which point the best chromosomes are outputted.

### Simulations

The simulated diploid genomes are first constructed by duplicating the human reference genome (NCBI build 37) into two sequences. Subsequently, SNPs, insertions, deletions, and inversions are randomly placed into the two sequences with various heterozygous rates and sizes (100-500 bp). The wgsim program [29] randomly generates paired-end reads from two homologous chromosomes with various insert sizes and error probabilities. Burrows-Wheeler Aligner (BWA) [30] then aligns short reads onto the assembled contigs. SAMtools and BCFtools determine the coordinate/alleles of heterozygous SNPs/indels [29]. The proposed SV detection module identifies other deletions, insertions, and inversions. Each site on the duplicated chromosome has a 0.01 SNP rate to alter the allele to the others. Generating and aligning paired-end reads from these diploid genomes produces standard SAM alignments.

**Fig 13:**
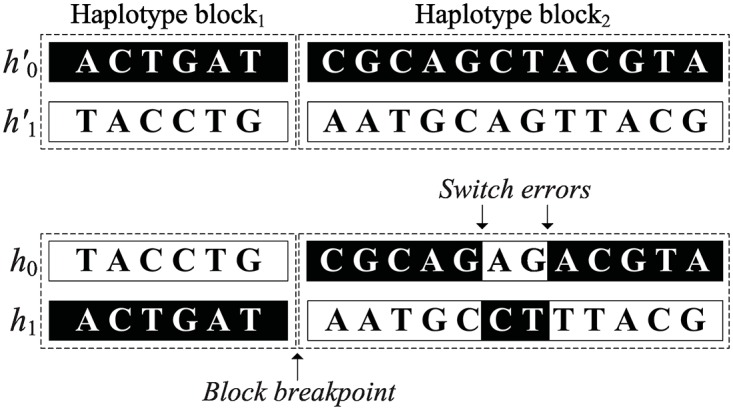
Illustration of *switch* *errors* and *block* *breakpoints*. In haplotype block_2_, there are two switch errors, where 1^st^ to 5^th^ bases, 8^th^ to 12^th^ bases are from $h_{0}^{\prime }$ but 6^th^ to 7^th^ bases come from $h_{1}^{\prime }$ on inferred haplotype *h*_0_.

This study defines haplotype assembly accuracy using a metric analogous to switching errors. However, this metric is able to reflect the fragmentation caused by discontinuous haplotype blocks. Specifically, given a real haplotype pair $H^{\prime }=\left(h_{0}^{\prime },h_{1}^{\prime }\right)$ and an inferred haplotype pair *H* = (*h*_0_, *h*_1_) within a haplotype block, a switch error represents that two adjacent haplotype segments, where one is from $h_{0}^{\prime }$ and the other is from $h_{1}^{\prime }$, are misjoined to form $h_{0}^{\prime }$ and $h_{1}^{\prime })$ (Fig 13). Denote *S* and *N* as the number of switch errors and total SNPs, respectively. The maximum possible *S* is thus *N* − 1. Define *B* as the number of haplotype block partitions within the assembled haplotypes. The switch errors purely caused by the assembly algorithm only occur at blocks with at least two SNPs, whereas a block with only one SNP has no need of a haplotype assembly. Therefore, the accuracy of the assembled haplotype pair *H* is $1-\frac{switch\;errors}{N-B-1}$ for haplotype blocks with at least two SNPs.

### BAC Sequencing

Two Bacterial Artificial Clone (BAC) libraries from a pilot sequencing of *Erycina pusilla* were constructed by randomly shearing the genomic DNA, which consists of sixty 100 kb BACs. These BACs were pooled and sequenced using the Illumina Genome Analyzer. A paired-end library of 300 bp insert size was constructed and sequenced up to 100bp read length. Potential contamination from E. Coli and vector sequences was cleaned by first aligning short reads onto the NCBI E. coli genome and NCBI VecScreen database (http://www.ncbi.nlm.nih.gov/VecScreen/VecScreen.html) using BWA, which was removed from the subsequent process. Only the clean paired-end reads were assembled by the HapSVAssembler pipeline.

## Results

The HapSVAssembler pipeline was implemented in C/C++, multithreaded, and encapsulated using bash script that supports standard formats as the input (e.g., fasta, SAM). The source code and program have been uploaed to GitHub (https://github.com/ythuang0522/HapSVAssembler). Various experiments were conducted to evaluate the assembly accuracy and contiguity of the HapSVAssembler. To the best of our knowledge, no existing assemblers are able to assemble haplotypes by using paired-end sequencing from NGS platforms. However, this study presents a comparison of the proposed method with two approaches proposed for Sanger sequencing. The first approach is called MaxSAT [18], and the other is called MEC/GA [28]. Both approaches attempt to separate single-end reads into paternal and maternal haplotypes with minimum error corrections. These programs are compared over various genome sizes, insert sizes (HapSVAssembler only), read lengths, sequencing coverage rates, and error rates.

### Assembly Accuracy and Contiguity

**Fig 14:**
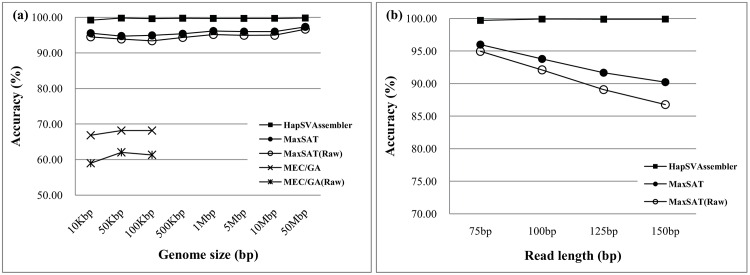
The accuracy for different genome size and read length. The paternal and maternal genomes differes in 1% SNPs. The mean insert size is 250bp with 25bp standard deviation, the sequencing coverage is 20X, and the sequencing error rate is 1%. (a) The accuracy for different genome sizes; (b) The accuracy for different read lengths.

Fig 14(a) shows the accuracies of genome sizes ranging from 10 kbp to 50 Mbp, where each data point represents the average of 10 data sets. The execution of MEC/GA takes longer than one day when the genome is larger than 500 kbp, which is not reported in the following experiments. The result indicates that the HapSVAssembler has significantly greater accuracy than MaxSAT and MEC/GA (marked by Raw). The partition of haplotypes into blocks in the proposed method is the major reason for this huge difference. The block partition breaks down the original assembly problem into smaller subproblems, which helps the algorithm find the optimum solution. To compare the underlying algorithms without the partition effects, we also manually partitioned the haplotypes into blocks, invoked the MEC/GA and MaxSAT separately for each block, and recomputed their accuracies. Although these measures improve the accuracies of both approaches, they are still much lower than that of the HapSVAssembler. Because the MEC/GA accuracy is much worse than the other two methods, the following comparative study omits its results. In view of the influence of read lengths to accuracy and completeness, longer reads are associated with a higher accuracy in the HapSVAssembler because the expected number of SNPs covered by one read fragment increases (Fig 14(b)). However, the accuracy of MaxSAT with a long read length drops unexpectedly.

**Fig 15:**
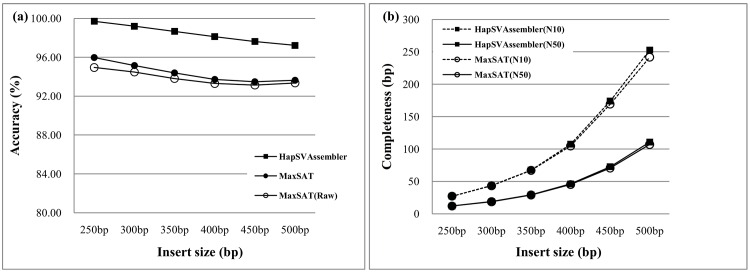
Assembly accuracy and contiguity for different insert size. The paternal and maternal genomes differes in 1% SNPs. The genome size is 5Mbp. The read length is 75bp and the sequencing coverage is 20X. The error rate of SNP/SV matrix is 1%. (a) The accuracy for different insert sizes *μ* with $\frac{\mu }{10}$ standard deviation; (b) The comparison of N10/N50 for different insert sizes.

**Fig 16:**
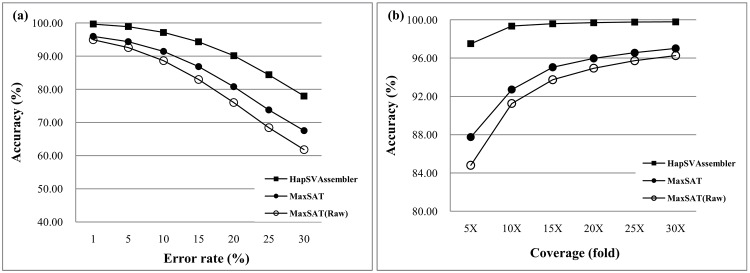
Assembly accuracy for different error rate and sequencing coverage. The similarity between diploid genome is 99%, and the genome size is 5Mbp. The read length is 75bp, and the mean of insert size is 250bp with 25bp standard deviation. (a) The accuracy for different error rate in SNP/SV matrix; (b) The accuracy for different sequencing coverage.

Most sequencing protocols support short and long inserts. Fig 15(b) plots the N10 and N50 of both approaches. The assembled contig N10 size of the HapSVAssembler is longer than that of MaxSAT, which does not consider the SVs. However, the tradeoff is a decrease in accuracy (Fig 15(a)). We also examined the influence of the HapSVAssembler on various error rates in the SNP/SV matrix. In erroneous data with a 25% error rate, the HapSVAssembler can still reconstruct haplotypes with an accuracy greater than 80%, and has a high tolerance for noise or errors (Fig 16(a)). Fig 16(b) shows that accuracy approaches 99% in 10X coverage, confirming its ability to achieve accurate results with a low experimental cost.

**Fig 17:**
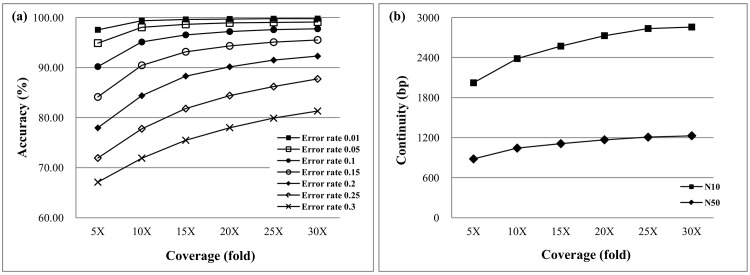
Assembly accuracy and contiguity for different sequencing coverage and error rates. (a) The accuracy higher than 90% can be obtained with low error rate simulations even in low coverage; (b) The comparison of N10/N50 for different sequencing coverage.

To identify the factors that most affect HapSVAssembler accuracy, Fig 17(a) plots the association between accuracy and different sequencing coverage rates according to various error rates. These results show that accuracy is always higher than 90% in low error rate simulations (error rate ≤ 0.1). The accuracy of high error rate data can be efficiently overcome by high sequencing coverage; for example, the accuracy of a 0.3 error rate simulation improves from ≈67% to ≈81% when the coverage increases from 5-fold to 30-fold. The error rate is a crucial factor influencing HapSVAssembler accuracy. Fig 17(b) shows the association between continuity and sequencing coverage. It is often expected that a higher coverage of sequencing should lead to more contiguous assembly (longer N10 and N50 simultaneously). However, this improvement is limited by the average distance between any two adjacent SNP/SVs, and N10/N50 gradually converges on 25-fold to 30-fold. Table 2 shows the running time for various genome sizes in three compared methods, where each datum represents the average of five independently simulated data. To accelerate the HapSVAssembler and MEC/GA, we separately used 10 and 16 threads to compute in parallel.

**Table 2:** Running time of HapSVAssembler, MaxSAT and MEC/GA.

Genome size	HapSVAssembler	MaxSAT	MEC/GA
10Kbp	0.004 seconds	0.400 seconds	33.800 seconds
50Kbp	0.240 seconds	0.600 seconds	33.673 minutes
100Kbp	0.680 seconds	0.600 seconds	3.801 hours
500Kbp	3.270 seconds	1.400 seconds	-
1Mbp	6.260 seconds	2.200 seconds	-
5Mbp	31.710 seconds	9.000 seconds	-
10Mbp	73.560 seconds	19.800 seconds	-
50Mbp	4.825 minutes	1.760 minutes	-

### Pilot Sequencing of a Diploid Genome

The HapSVAssembler was tested on a *de novo* pilot sequencing of the *Erycina Pusilla* genome, which is expected to be highly heterozygous yet a good model genome due to short life cycle. A BAC library (representing 5MB of the diploid genome) were constructed and sequenced using the Illumina HiSeq with a read length of 100 bp and an insert size of ≈300 bp. The assembled contigs sum up to 4.7Mb with N50 = 12kbp. The results (Table 3) indicate that HapSVAssembler identified 12,781 heteozygous SNPs and 573/29 hemizygous insertions/deletions differing between paternal and maternal genomes. The insertions and deletions sum up to 72,896bp and 5,080bp, respectively. On average, The sizes of insertions and deletions are 127bp and 175bp, respectively. Overall, the heterozygosity of the partial genome (including SNPs and SVs) is about 1.92% (90,713bp/4,705,947bp), which implies the subsequent whole genome assembly will be very challenging. Although the number of SVs are much less than that of SNPs, the genomic regions occupied by SVs are much larger than that of SNPs (77,976bp vs 12,781bp), which implies the degree of heterozygosity computed from heterozygous SNPs or from the *k*-mer spectrum might be under-estimated. On the other hand, the proposed method is able to precisely compute the heterozygosity regions across various types of variations.

**Table 3:** Heterozygous variations, including heterozygous SNPs and hemizygous insertions/deletions/inversions, detected during assembly of diploid genome.

	SNPs	Insertions	Deletions	Inversions
Number	12781	573	29	0
Min Size	1bp	100bp	100bp	-
Max Size	1bp	337bp	363bp	-
Mean Size	1bp	127bp	175bp	-
Total Size	12,781bp	72,896bp	5,080bp	0bp
Genome Percentage	0.27%	1.55%	0.1%	0%

### Convergence Rate of GA

**Fig 18:**
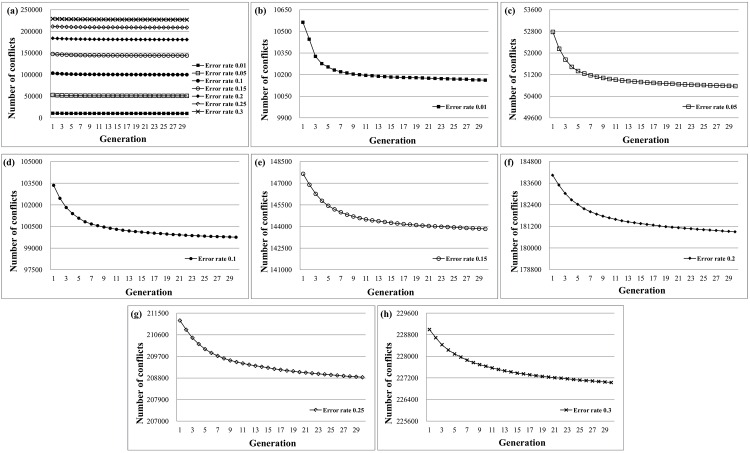
In-time behavior of proposed GA for different error rates. The best fitness value (number of conflicts) of first thirty generations in different error rate from 0.01 to 0.3.

**Fig 19:**
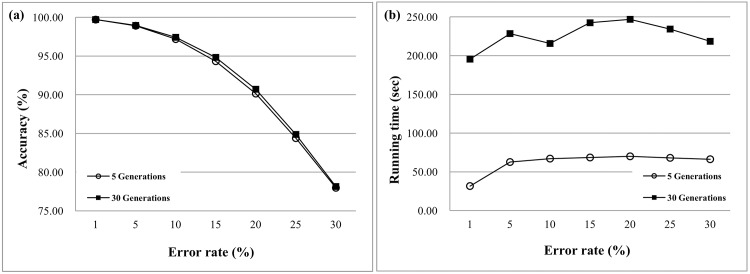
The accuracy and running time of different generations in GA. (a) the accuracy in different error rates with 5 and 30 generations; (b) the running time in different error rates with 5 and 30 generations.

This section investigates the convergence of solutions and the reduction of problem size in the proposed GA. Fig 18 shows the best fitness value of the first 30 generations at error rates ranging from 0.01 to 0.3. Results show that the fitness values often converge in the first 5 to 10 generations for low error rates because the heuristic initialization collects good solutions at the beginning of the evolution. Therefore, the HapSVAssembler avoids many random steps to reduce the computational time and stochastic search. Fig 19(a) shows the accuracy in different error rates with 5 and 30 generations. The accuracy advantage of 30 generations compared to 5 generations is limited. However, the running time increases drastically (Fig 19(b)). Given the limited advantage but much higher computational cost of 30 generations, the default setting of the HapSVAssembler was set to only five generations.

**Fig 20:**
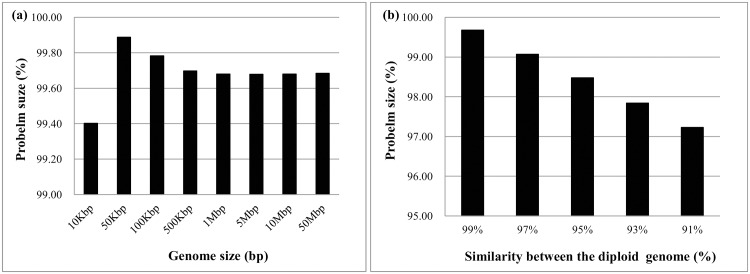
The percentage of reducted problem sizes of CMEC model. (a) Under 99% similarity between the diploid genome, 0.3% of reads can be constrained together; (b) Problem size is decreased when the difference between the diploid genome is increased.

The problem size can be reduced by the hard constraints in the CMEC formulation. Thus, Fig 20(a) shows the percentage of constrained read fragments with respect to the genome size. Results show that only 0.3% read fragments can be constrained together under 99% similarity. The best reduction percentage of problem size occurs at a 10 kbp genome size because the SVs are un-proportionally created in small genome size (e.g., 10 kbp and 50 kbp). Fig 20(b) shows that the problem size consistently decreases with respect to the increasing divergence between diploid genome in that more reads are constrained by the heterozygous variants between the diploid. In summary, the CMEC formulation reduces the problem size in a GA at a higher coverage rate and for larger genomes.

## Discussion

The error rate of Illumina sequencers is known to be non-uniform. As a consequence, the accuracy of breakpoint reads (and thus SV calling) might be reduced since alignment is less reliable at these error-prone or repetitive regions. Below We discussed the influence of error bias and repeats on our algorithm separately under the re-sequencing and de novo assembly scenarios. If a fully-assembled genome is available, the error rate of breakpoint reads indeed may elevate at high-GC/repeat boundaries. However, in addition to breakpoint reads, discordant reads (e.g., abnormal mapping distance w.r.t. insert size) are also included in the prediction, which are less affected by the non-uniform error bias. Therefore, users may improve the specificity by requiring both sufficient discordant and breakpoint reads when calling SVs, although this would sacrifice sensitivity. It look to us a better solution to this problem may be inclusion of sequence context/motif of these error-biased regions (e.g., GGC motif or GC density) into the SV calling algorithm, in addition to the conventional breakpoint/discordant reads. Furthermore, we feel this problem might become a minor issue if the third-generation sequencers are used instead (e.g., PacBio or Nanopore), which produce less GC bias and longer reads for spanning repeats. On the other hand, if a fully-assembled genome is unavailable and de novo assembly is required, our algorithm is less affected by this error-biased problem. These error-biased/repetitive regions reduce not only the alignment accuracy but also the assembly contiguity. As a consequence, most contigs are only assembled upto boundaries of these error-biased/repetitive regions. In other words, our algorithm is in fact tested on the contigs in which the majority do not contain these problematic regions.

The current implementation does not support multiple libraries, because the inclusion of SV constraints from multiple libraries into the CMEC formulation will generate a complex optimization problem, whereas conflicting constraints derived from different libraries would prevent search of feasible solutions. The major output file has a format similar to the conventional VCF file yet including haplotype block boundaries and SV alleles (e.g., insertion or deletion). We also provided another output file similar to fasta yet containing the paternal and maternal haplotype sequences separated by block boundaries. Other output files mainly provide the loci and allele information of SNPs and SVs and details can be found on README on GitHub.

## Supporting Information

S1 Fig The software components and flowchart of HapSVAssembler. The short reads are first aligned to the assembled genome. Subsequently, SNPs and SVs are identified and used to construct a SNP/SV matrix. Finally, the paternal and maternal haplotypes are separated in order to reconstruct the diploid genome.
(TIF)
