# Supplementary figures and images for: A Genetic Algorithm for Diploid Genome Reconstruction Using Paired-End Sequencing
Source: PLoS One. 2016 Nov 18;11(11):e0166721. doi: 10.1371/journal.pone.0166721 (PMC5115803; doi:10.1371/journal.pone.0166721)

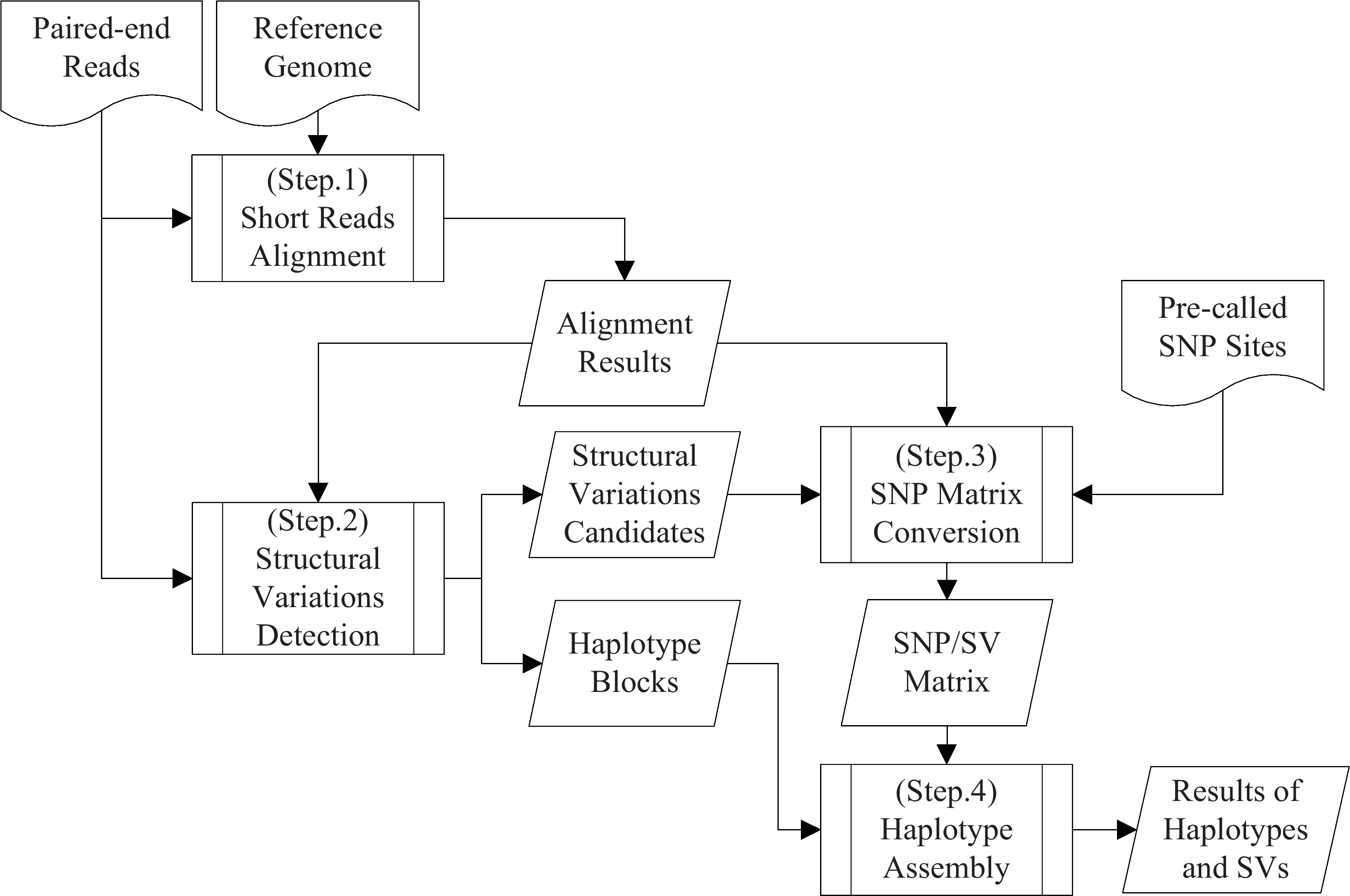

Supplement: S1 Fig — The short reads are first aligned to the assembled genome. Subsequently, SNPs and SVs are identified and used to construct a SNP/SV matrix. Finally, the paternal and maternal haplotypes are separated in order to reconstruct the diploid genome. (TIF) [file pone.0166721.s001.tif]
